# FACTORS THAT PREDICT LONELINESS FOR MIDDLE-AGED AND OLDER ADULTS DURING THE COVID-19 PANDEMIC

**DOI:** 10.1093/geroni/igac059.1694

**Published:** 2022-12-20

**Authors:** Angela Curl, Katie Wolf

**Affiliations:** Miami University, Oxford, Ohio, United States; Miami University, Oxford, Ohio, United States

## Abstract

The Protection Motivation Theory (Eberhardt & Ling, 2021; Rogers, 1975) describes factors that influence intention and engagement in preventive health behaviors, including knowledge/experience, threat appraisal, and coping/efficacy appraisal. Public health responses to the covid-19 pandemic were designed to increase knowledge, emphasize potential severity of being infected, and promote preventive health behaviors – all with the goal of reducing infection transmission. However, these efforts may have inadvertently increased loneliness, particularly for older adults. This study used 2020 Health and Retirement Study data (N=1,687 adults over age 50) to examine predictors of loneliness based on the PMT framework, controlling for demographic factors and 2016 loneliness scores. Structural Equation Model results indicate that being at higher risk for covid-19 complications and death was associated with lower feelings of control over health (B=-.09), greater likelihood of knowing someone who died from covid-19 (B = .20), and higher overall concerns about covid-19 (B=.12), all p<.01. Higher concerns about covid-19 and knowing someone who died from covid-19 were associated with more protective behaviors (B=.41, p<.01; B=.05, p<.05; respectively). Not knowing someone who died of COVID-19, and lower perceived control over health and social life were all significantly associated with higher loneliness scores. These results suggest that interventions that promote perceived control over one’s health and social life may be effective in reducing feelings of loneliness in this population, and that feelings of control over health also increases preventive health behaviors that reduce the risk of covid-19 infection.

